# Epidemiological survey of intentional poisoning suicide during 1993-2013 in Ilam Province, Iran

**DOI:** 10.1186/s12889-016-3585-9

**Published:** 2016-08-30

**Authors:** Yosra Azizpour, Khairollah Asadollahi, Kourosh Sayehmiri, Satar Kaikhavani, Ghobad Abangah

**Affiliations:** 1 Department of Clinical Epidemiology, Faculty of Health, Ilam University of Medical Sciences, Ilam, Iran; 2 Department of Social Medicine, Faculty of Medicine, Ilam University of Medical Sciences, Ilam, Iran; 3 Psychosocial Injuries Researches Center, Ilam University of Medical Sciences, Ilam, Iran; 4 Department of Clinical Psychology, Faculty of Medicine, Ilam University of Medical Sciences, Ilam, Iran; 5 Department of Internal Medicine, Faculty of Medicine, Ilam University of Medical Sciences, Ilam, Iran

**Keywords:** Suicide, Method of suicide, Intentional poisoning, Ilam, Iran

## Abstract

**Background:**

Suicide is an important social tragic phenomenon which occurs by different tools or methods in different communities. Considering deliberate poisoning as a common and important method in Ilam province for suicide, the present study was launched to epidemiologically assess committing suicide in Ilam province, Iran, based on intentional poisoning.

**Methods:**

By a retrospective study, all the recorded data associated with intentional poisoning suicide in Ilam Province during 1993–2013 were analyzed. All the demographic variables and the suicides’ outcomes were analyzed using the Chi-Square test, and the univariate and multivariate logistic regression models.

**Results:**

Totally, 6794 cases of suicide (annual incidence rate of 87.28/ 100, 000) associated with poisoning were evaluated. The incidence rate of suicide attempts was 84.63/ 100, 000 (94.51 in female and 74.98 in male) and the incidence rate of completed suicide was 2.17/ 100, 000 (1.94 in female and 2.40 in male). Also, the highest rates of attempted and completed suicide (annual incidence rate of 172.42 and 4.14, respectively) were attributed to the age group of 15–24 year.

**Conclusion:**

Females had a greater tendency to commit suicide by poisoning, and the lower level of education, the age group of 15–24 years and single individuals were more associated with suicide using poisonings. The incidence of attempted suicide in females and completed suicide in males was higher in this method. Considering the high rate of suicide by poisoning, further attention and supervision on the sale and reserve of drugs and poisons is necessary.

Meanwhile, it seems that due to momentary emotions during the pubertal stage, the risk of committing suicide is increased especially among unemployed individuals; therefore, performing an extensive psychotherapy intervention is needed in the societies with younger populations.

## Background

Completed suicide is defined as a deliberate action of self-damage resulting in death of an individual; however, an attempted suicide is an action through which the individual harms, but not kills him/her self [1] and is mostly a pretention to incite other people’s feelings. According to the fact sheet published by WHO, over 800 thousand people die from suicide annually worldwide and it is the second leading cause of death among people aged between 15 and 29 years old [2]. There are several ways through which an individual chooses a method of suicide, and of course, the prevalence rates of these methods vary by geographical regions. Ajdacic-Gross et al. reported that the most common method of suicide in Latin America and most Asian countries was intentional poisoning with pesticides [3], and in the United States of America 0.3 % of male and 0.5 % of female, and in the Republic of Korea (South Korea) 37.5 % of male and 42.8 % of female, have used pesticides to commit suicide [3]. In the same study, intentional drug poisoning was listed as one of the common methods of suicide in Britain and Nordic countries [3]. Morovat-Dar et al. indicated that, following hanging, poisoning is the leading cause of suicide in countries located at the Eastern Mediterranean regions [4].

In a meta-analysis study of suicide from 1981–2007 in Iran, the distribution of suicide methods were as follow: drugs (65 %), poisoning (12 %), self-immolation (15 %), hanging (9.1 %) and use of firearms (6 %) [5]. Based on health system database in Iran during 2001–2007, three common methods of suicide were as follow: Drug overdose (73 %), poisoning (10.6 %) and self-immolation (4.1 %) [6]. In the current study we considered drugs as parts of chemical or poisonous substances and therefore suicide via poisoning ranked first in this province. Because deliberate poisoning was a common and important method of suicide in Ilam province, therefore, the present study focused on suicide based on intentional poisoning. There is a detailed and comprehensive system of registration for suicide in Ilam province and all complete and incomplete suicides are recorded in this system confidentially and they only can be used by authorized people for academic or interventional purposes.

Depending on the various internal and external factors, people use different tools or methods to commit suicide. These factors can be associated with ethnical, religious, social and cultural status, along with the availability of the tools which vary across different regions [7].

The present study aimed to assess the incidence rate of intentional poisoning in Ilam province and the influential factors behind the selection of that particular type of suicide method.

## Methods

### Study design

The present study was a retrospective investigation including all the individuals who committed suicide between 1993 and 2013 in Ilam province, west of Iran. The required data, which was recorded in a comprehensive system of suicide registration, was obtained from the University of Medical Sciences in Ilam. The data used in the current study was not openly available for public and it was only accessible for authorized people.

Ilam Province is one of the 31 provinces of Iran. It is located in the west part of the country, bordering Iraq including an area of 19,086 square kilometers and its population is approximately 560,000 people based on the last Iranian National Census in 2012.

All individual data of suicide were already interred in an access database (a comprehensive system of suicide registration in Ilam University of medical sciences) and then we transferred them to an Excel file, then we used specific variables including demographic data including age, gender, marital status, level of education, occupational status, place of life and city of residence; the date of occurrence of suicide (in terms of year, season, month, and day time); outcome of suicide (attempted or completed), and other variables such as motives of suicide, housing conditions, family structure, racial status, and the status of committing suicide. Finally, desired data was extracted and analysed by researchers. We used census data and there was not any sampling in data collection.

According to the national population census, conducted in year 2011, and based on the different variables, the incidence rate of suicide was calculated. Also, the specific incidence rate of the population at risk (>15 years) was calculated based on some variables.

### Statistical analysis

Chi-Squared and logistic regression tests are widely used in epidemiological studies regarding suicide analysis [8]. The Chi-Squared test was applied in order to achieve a significant difference regarding the frequency of epidemiological variables and the methods related to suicide. Also, in order to estimate the probability of death due to suicide as well as the matched probability ratio based on the considered variables, the univariate and multivariate logistic regression model were respectively used along with “Forward Likelihood method”. In a multivariate logistic regression model, several methods could be applied during analysis by SPSS software including: Enter, Forward Conditional, Forward Likelihood, Forward Wald, Backward Conditional, Backward Likelihood and Backward Wald and we have chosen Forward Likelihood model. In this method the first variable shows the highest correlation with depended variable entered into the model followed by the second variable with the highest correlation and etc consecutively. In all cases, *p* <0.05 was considered as significant.

This study was approved by the ethics committee of Ilam University of Medical Sciences and its relevant institutional ethics number was“ir.medilam.rec.1394.51”.

## Results

Totally, 6794 cases of suicide (annual incidence rate: 87.28/ 100, 000) due to poisoning were recorded during the study period. Among these cases, 6588 (annual incidence rate: 84.63/ 100, 000) were associated with the suicide attempts and 169 (annual incidence rate: 2.17/ 100, 000) belonged to completed suicide. The outcome of 37 suicide events was unknown.

**Fig. 1:**
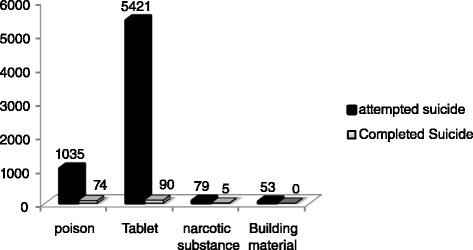
Frequency of different methods applied for intentional poisoning in Ilam province during1993-2013

Regarding to the methods used for poisoning, the highest frequency rate belonged to tablets among either completed or attempted suicides, and the lowest frequency rate was attributed to narcotics among only completed suicides (Fig. 1).

The maximum incidence rate of attempted suicide (94.5/100,000) and the maximum incidence rate of completed suicide (2.40/100,000) were seen among female and male, respectively. Also, the maximum incidence rate of attempted and completed suicide (172.42 and 4.14/100,000 respectively), were seen in the age group of 15–24 years. The Chi-Squared test showed a significant difference between suicides based on the age groups and gender (*P* < 0.05). According to the rest of demographic variables, the highest incidence rate of attempted and completed suicides was seen among single individuals. The highest (140.47/100,000) and the lowest (29.46/100,000) incidence rates of attempted suicide were seen among individuals with secondary education level and illiterate individuals, respectively. Also, the highest incidence rate of completed suicides (4.87/100,000) was seen among individuals with secondary education level. The maximum incidence rate of attempted and completed suicide (295.5 and 6.2/100,000 respectively), was seen among unemployed individuals. Also, the minimum incidence rate of attempted and completed suicide (13.03 and 0.68/100,000 respectively), was seen in farmers and office workers. In terms of place of life, those residing in the rural areas had the highest rate of attempted and completed suicide. The highest rates of attempted and completed suicide were identified in Dareshahr County (154.56 and 5.40 /100,000 respectively). According to the Chi-Squared test, there was a significant relationship between marital, educational or occupational status and place of life, with committing suicide (*P* < 0.05) (Table 1).

**Table 1:** Frequency and annual incidence rate of suicides per 100, 000 people in based on demographic characteristics in Ilam province during1993-2013

Variables		Attempted suicide		Completed suicide		*p*-value
		No. (%)	Annual incidence rate	No. (%)	Annual incidence rate	
Gender	Male	2939(96.9)	74.98	94(3.1)	2.40	0.004
	Female	3649(98)	94.51	75(2)	1.94	
Age	10–14years	143(98.6)	20.21	2(1.4)	0.28	0.0001
	15–24year	4001(97.7)	172.42	96(2.3)	4.14	
	25–34years	1788(98)	82.87	36(2)	1.67	
	35–44years	411(96)	29	17(4)	1.2	
	45–54years	152(96.2)	17.88	6(3.8)	0.71	
	55–64years	51(92.7)	9	4(7.3)	0.71	
	≥65 years	37(82.2)	7.83	8(17.8)	1.69	
Marital status	Single	4008(97.5)	137.51	101(2.5)	3.47	0.001
	Married	2532(97.5)	56.15	64(2.5)	1.42	
	Divorced and Widow	24(85.7)	6.73	4(14.3)	1.12	
level of education	Illiterate	435(94.8)	29.46	24(5.2)	1.63	0.0001
	Elementary	551(96.2)	49.33	22(3.8)	1.97	
	Secondary	1240(96.6)	140.47	43(3.4)	4.87	
	High school and Diploma	3278(98.3)	137.46	56(1.7)	2.35	
	University educated	759(98.4)	42.88	12(1.6)	0.68	
Occupation status	Housewife	1581(98.1)	71.17	30(1.9)	1.35	0.0001
	Unemployed	2291(97.9)	295.54	48(2.1)	6.19	
	Self-employed	526(97.2)	117.87	15(2.8)	3.36	
	Office worker	173(97.2)	23.4	5(2.8)	0.68	
	Farmer	109(86.5)	13.03	17(13.5)	2.03	
	Student	1308(97.6)	70.66	32(2.4)	1.73	
	Worker	128(96.2)	45.54	5(3.8)	1.78	
place of life	Urban	3544(98.3)	71.05	61(1.7)	1.22	0.005
	Rural	2001(97.2)	72.03	57(2.8)	2.05	
City of Residence	Abdanan	531(98.3)	81.62	9(1.7)	1.38	0.087
	Ilam	2615(97.9)	87.38	57(2.1)	1.9	
	Ayvan	942(98)	134.46	19(2)	2.71	
	Dareh-shahr	1289(96.6)	154.56	45(3.4)	5.4	
	Dehloran	349(98.9)	38.31	4(1.1)	0.44	
	Shirvan-Chardavol	585(97.2)	57.84	17(2.8)	1.68	
	Malekshahi	89(96.7)	28.91	3(3.3)	0.97	
	Mehran	130(96.3)	34.55	5(3.7)	1.33	

Chi-squared test showed a significant relationship between months of suicide and the outcome of suicide events; according to which the highest attempted and completed suicides’ proportions (99 and 3.8 %) were identified in December (478 out of 483) and January (21 out 541), respectively.

Based on the univariate logistic regression model, the probability of death occurrence by suicide in males (OR = 1.55; 95 % CI: 1.14–2.11; *P* = 0.005) was significantly higher than that in females. The age group of 55–64 and ≥65 years (mortality rate of 5.61 and 15.46, respectively) showed a significantly higher death rate due to suicide compared to that among the age group of 10–14 years. The probability of death due to suicide was higher among the divorced-widows and illiterate individuals. A farmer was 8.2 times more likely to die due to suicide than a housewife. Also, the probability of death due to suicide among villagers was 1.65 times more than that among those living in urban areas (Table 2).

**Table 2:** The estimated probability of death from suicide, according to demographic variables using univariate logistic regression analysis in Ilam province1993-2013

Variables		Odds ratio	95 % CI	*p*-value
Gender	Male	1.55	1.14–2.11	0.005
	Female^a^	1.0		
Age	10–14years ^a^	1.0		
	15–24year	1.72	0.42–7.02	0.453
	25–34years	1.44	0.34–6.04	0.618
	35–44years	2.96	0.67–12.96	0.150
	45–54years	2.82	0.56–14.21	0.208
	55–64years	5.61	1.00–31.54	0.050
	≥65 years	15.46	3.15–75.90	0.001
Marital status	Single^a^	1.0		
	Married	1.003	0.73–1.38	0.985
	Separated and Widow	6.61	2.53–19.41	0.001
level of education	Illiterate	3.49	1.73–7.04	0.0001
	Elementary	2.52	1.24–5.15	0.011
	Secondary	2.19	1.14–4.19	0.017
	Diploma	1.08	0.58–2.02	0.809
	University educated^a^	1.0		
Occupation status	Housewife^a^	1.0		
	Unemployed	1.10	0.70–1.75	0.667
	Self-employed	1.50	0.80–2.82	0.202
	Office worker	1.52	0.58–3.98	0.389
	Farmer	8.22	4.40–15.37	0.0001
	Student	1.29	0.78–2.13	0.320
	worker	2.06	0.79–5.40	0.141
place of life	Urban^a^	1.0		
	Rural	1.65	1.15–2.38	0.007
City of Residence	Abdanan^a^	1.0		
	Ilam	1.29	0.63–2.61	0.487
	Ayvan	1.19	0.53–2.65	0.670
	Dareh-shahr	2.06	1.00–4.24	0.050
	Dehloran	0.68	0.21–2.21	0.518
	Shirvan-Chardavol	1.71	0.76─.88	0.196
	Malekshahi	1.99	0.53─7.49	0.309
	Mehran	2.27	0.75─6.88	0.148

^a^Reference

Based on multivariate logistic regression model and after adjusting on confounder variables, the probability of death due to suicide in illiterate people was 4.18 times more than that among those with academic education, which was statistically significant. Also, the probability of complete suicide occurrence in farmers was significantly higher compared to that in housewives (OR = 7.35; 95 % CI: 3.02–17.90). The probability of death occurrence among divorced and widowed individuals was 6.36 % and the chance of death was 1.52 times more likely to happen in January compared to March (Table 3).

**Table 3:** The relationship between variables in probability of death from suicide according to multivariate logistic regression analysis in Ilam province during1993-2013

Variables		Adjusted odds ratio(95 % CI)	*p*-value
Level of education	Illiterate	4.18(1.58–11.05)	0.004
	Elementary	3.06(1.20–7.79)	0.019
	Secondary	2.40(1.05–5.50)	0.038
	Diploma	1.22(0.55–2.71)	0.619
	University educated^a^	1.0	
Occupation status	Housewife^a^	1.0	
	Unemployed	1.80(0.83–3.87)	0.136
	Self-employed	2.43(1.06–5.60)	0.035
	Office worker	3.42(1.07–10.97)	0.039
	Farmer	7.35(3.03–17.90)	0.0001
	Student	2.45(1.05–5.76)	0.038
	Worker	2.57(0.81–8.13)	0.107
Marital status	Single^a^	1.0	
	Married	0.97(0.54–1.75)	0.935
	Separated and Widow	6.36(1.29–31.5)	0.023
Month of suicide	March	1.31(0.53–3.25)	0.556
	April	1.27(0.55–2.95)	0.572
	May	0.45(0.16–1.30)	0.141
	June	1.20(0.50–2.88)	0.675
	July	0.69(0.26–1.82)	0.456
	August	0.34(0.11–1.04)	0.060
	September	0.66(0.25–1.74)	0.404
	October	0.71(0.24–2.05)	0.529
	November	0.67(0.23–1.92)	0.461
	December	0.10(0.01–0.81)	0.032
	January	1.52(0.64–3.59)	0.344
	February^a^	1.0	

^a^Reference

There was not a significant relationship between the season of suicide and the outcome of suicide events. The highest rates of attempted and completed suicides (98.2 and 2.9 %) were occurred respectively in the summer (1891 out of 1926) and autumn (47 out 1612). Univariate regression analysis indicated that the probability of death due to suicide was decreased in the summer season compared to other seasons (OR = 0.67; 95 % CI: 0.43–1.06), but was increased slightly in the autumn and spring (Table 4).

**Table 4:** The frequency and odds ratio of death from suicide by other variables in the study in Ilam province, 1993–2013

Variables		Attempted suicide	Completed suicide	Odds ratio(95 % CI)	*p*-value
		No. (%)	No. (%)		
Season	Spring	1630(97.3)	45(2.7)	1.007(0.66–1.55)	0.159
	Summer	1891(98.2)	35(1.8)	0.67(0.43–1.06)	
	Autumn	1565(97.1)	47(2.9)	1.095(0.71–1.67)	
	Winter^a^	1495(97.3)	41(2.7)	1.0	
Motive of suicide	Psychic problem^a^	1401(97.4)	38(2.6)	1.0	0.215
	Physical problem	50(96.2)	2(3.8)	1.47(0.34–6.28)	
	Economical problem	77(93.9)	5(6.1)	2.39(0.91–6.25)	
	Family conflict	2662(97.1)	79(2.9)	1.09(.74–1.62)	
	Educational problem	84(97.7)	2(2.3)	0.88(0.21–3.70)	
	Addiction problem	59(92.2)	5(7.8)	3.12(1.19–8.23)	
	Unemployed problem	109(97.3)	3(2.7)	1.02(0.30–3.34)	
Housing condition	Personal^a^	1365(97.4)	36(2.6)	1.0	0.282
	Organizational	73(94.8)	4(5.2)	2.07(0.72–5.99)	
	Gratuitous	2185(96.4)	81(3.6)	1.41(0.94–2.99)	
	Rental	670(97)	21(3)	1.19(0.69–2.05)	
Family structure	Nuclear family^a^	2986(96)	123(4)	1.0	0.499
	Extended family	345(95.3)	17(4.7)	1.19(0.71–2.01)	
Status of committing suicide	Alone^a^	3162(96.1)	130(3.9)	1.0	0.860
	At the presence of others	132(96.4)	5(3.6)	0.92(0.37–2.90)	
Racial status	Lor^a^	704(96.2)	28(3.8)	1.0	0.946
	Kord	2628(96)	109(4)	1.04(0.68–1.59)	
	Others	32(97)	1(3)	0.79(0.10–5.96)	
Day Time of suicide	Morning	435(94.4)	26(5.6)	1.16(0.61–2.20)	0.964
	Noon	268(95)	14(5)	1.01(0.48–2.11)	
	Evening	868(94.9)	47(5.1)	1.04(0.46–1.88)	
	Night^a^	310(95.1)	16(4.9)	1.0	

^a^Reference

Based on the motives of suicide variable, the highest frequency of attempted suicides was attributed to educational problems (97.7 %; 84 out of 86 cases) followed by psychological problems (97.4 %; 1401 out of 1439 cases), and unemployment problems (97.3 %; 109 out of 112 cases). Among those with completed suicides, the addiction problems (7.8 %; 5 out of 64 cases) showed the highest percentage followed by economic problems (6.1 %; 5 out of 82 suicides) (*P* > 0.05). The univariate logistic regression analysis showed that addiction can significantly increase the probability of death due to suicide (OR = 3.06; 95 % CI: 1.16–8.08) (Table 4). In terms of housing conditions, the probability of completed suicide was 2.07 times higher among those living in houses provided by the government compared to those living in private houses. The probability of death due to suicide was 1.19 times higher among extended families compared to those in nuclear families. Those who committed suicide lonely experienced an increase in the probability rate of death due to suicide. Also, the highest frequency of completed suicide occurred in the mornings (26 out of 461 cases, 5.6 %) (Table 4).

**Fig. 2:**
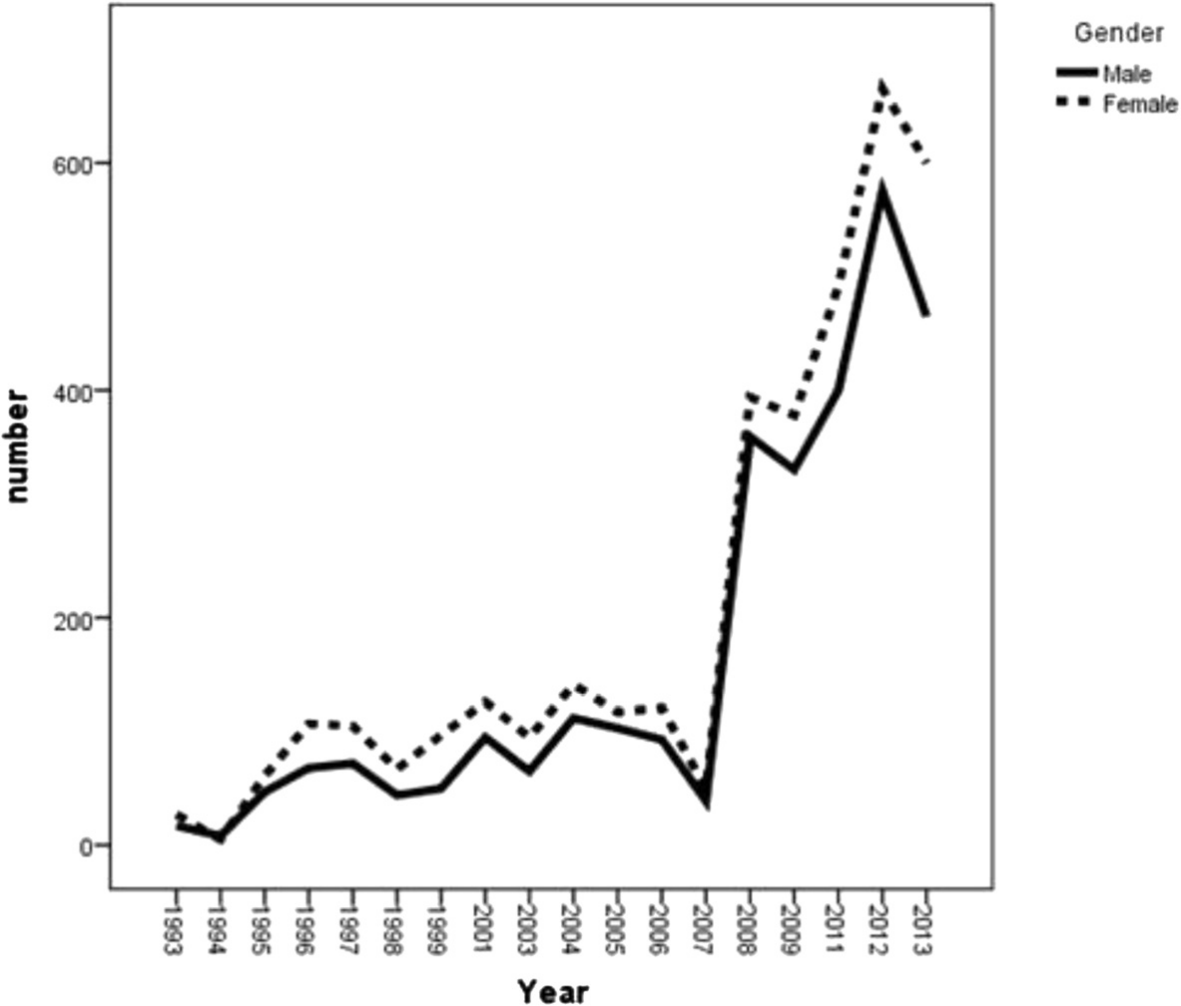
Trend of attempted suicide by poisoning, according to gender in Ilam province during1993-2013

**Fig. 3:**
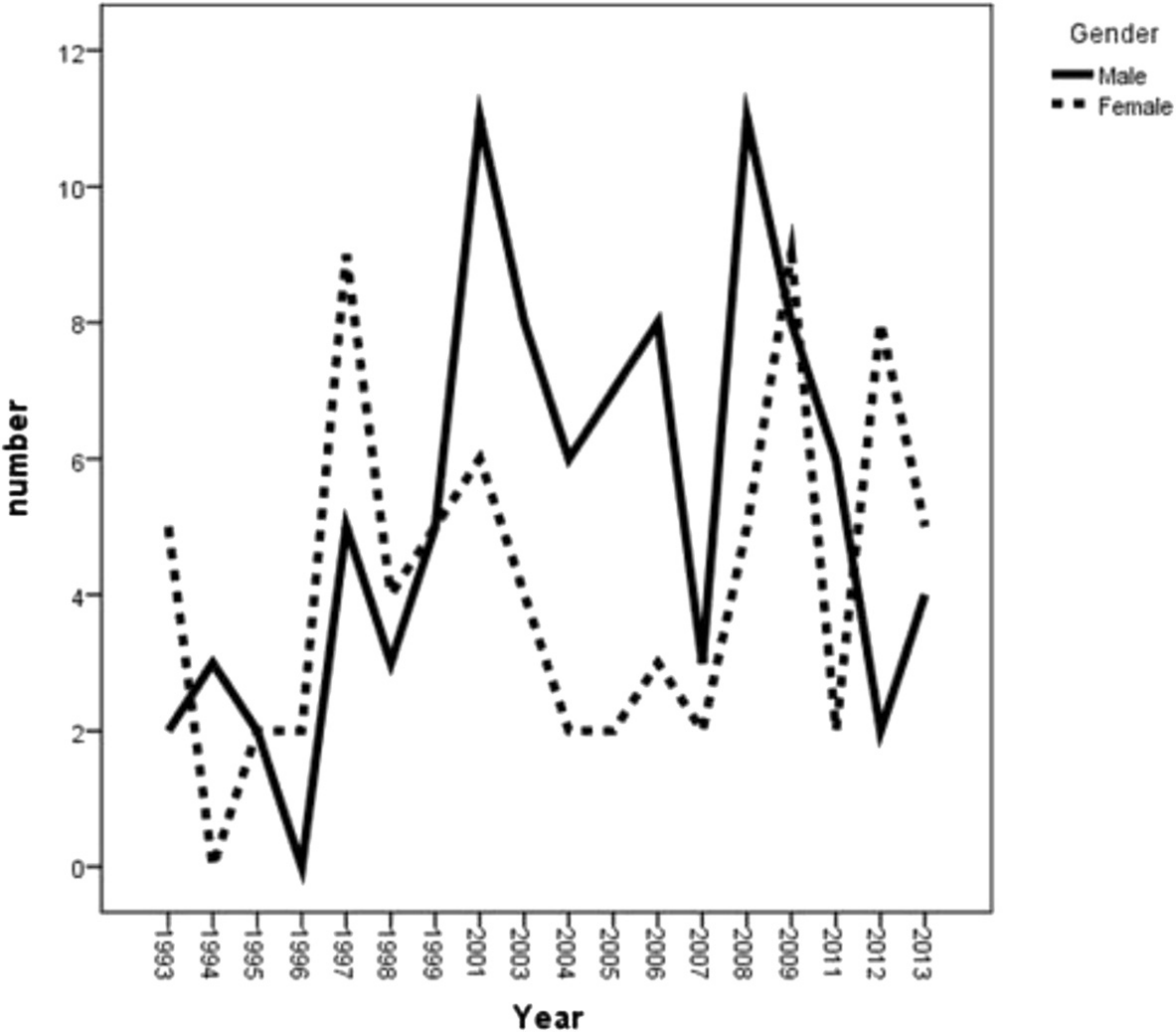
Trend of completed suicide by poisoning, according to gender in Ilam province during1993-2013

**Fig. 4:**
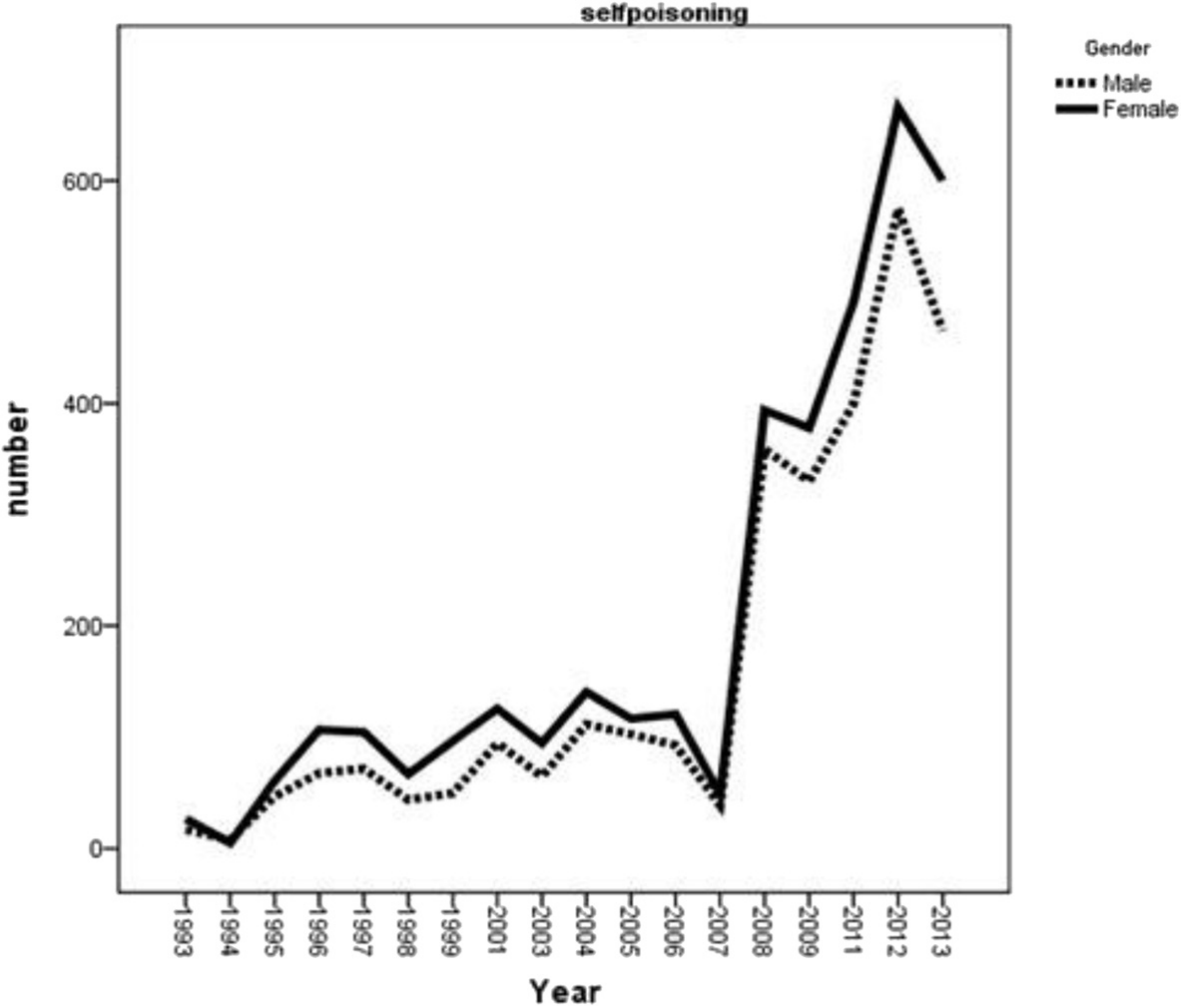
Trend of crude suicide by poisoning, according to gender in Ilam province during1993-2013

There was an increasing trend for attempted suicides during 1993–2013 with a very sharp rising trend from year 2007 onward either for men and women. However, the amount of attempted suicide was higher in females compared to males (Fig. 2). The incidence rate of completed suicide showed a decreasing trend among males during the years of 2008–2012 which increased again during 2012–2013. This increasing trend was the same for females during 2011–2012 which decreased again during the year 2013 (Fig. 3). In general, the trend of crude suicide by poisoning showed that females are more likely to have suicide compared to males (Fig. 4).

## Discussion

The current study revealed that the occurrence rate of suicide attempts using intentional poisoning in Ilam province was 87.28/100,000, which is relatively high compared to those reported from other areas of the country. Najafi et al investigated the occurrence of suicide due to poisoning in Kermanshah province, located at the neighborhood of Ilam province, and reported an incidence rate of 153/100,000 [9] which was even higher than that reported in the present study. The high occurrence rates in both studies may be attributed to the cultural and geographical similarities between these two provinces. Among the 8611 suicide occurrence in Ilam province, during the study period, 6794 cases were due to intentional poisoning; indicating that poisoning is the leading method of suicide in this province. Studies in other areas of the country, such as Golestan (North East of Iran) [10], Kashan (center of Iran) [7], Arak (center of Iran) [11], and Hamadan (West of Iran) [12], also reported poisoning as the leading method of suicide. Studies from some other Asian countries, such as India [13, 14], South Korea [15], Taiwan (during years 1970–1980) [16], Pakistan [17], also identified poisoning as the most common method of suicide. In a study conducted in Scotland, it was reported that the most common method of suicide among females was intentional poisoning but it was the second common method among males after hanging method [18]. In a similar study in Finland, intentional poisoning was ranked as the first among the non-violent methods of suicide [19]. Our findings were almost in accordance with other reports from Iran and other societies worldwide, particularly those from Asian countries.

Worldwide, 30 % of suicides are committed by pesticides and the use of this method; however, varies markedly from 4 % in Europe to more than 50 % in the Pacific region [20]. These differences are believed to be, in part, due to availability of the different methods [21], and the social and cultural differences between communities and the same concept could be considered for the method of poisoning. Of course, the difference in using this method of suicide in Ilam (Iran) compared with Asian and European countries depends on the accessibility of the suicide tools, to the difference of cultures and social conditions of these communities.

Among the intentional poisoning methods, tablet followed by poisoning were the most prevalent methods of suicide in our study. In some other studies conducted in different parts of Iran [6, 22, 23], drug followed by poisoning were the most frequent methods used for suicide which were in accordance with our results. In some studies conducted in the rural areas of China, Serilnaka, Thailand, and Malaysia, poisoning, with a frequency of >65 %, was the most common method of suicide [24]. In the meta-analysis conducted in Iran, it was also reported that venenation using drugs (75 %) and agricultural poisons (13 %) were the most frequent methods of poisoning [25], which can be attributed to the availability and ease of access to different drugs and poisons.

### Gender

Attempted suicide in females and completed suicide in males showed the highest incidence rate, with a significant difference for gender. According to Zyoud et al., suicide using acetaminophen was higher among women compared to men in Malaysia, but men used more poisonous doses of this drug [26]. In Morocco, Mahir et al. reported a correlation between progress toward recovery in women, and progress toward death in men after committing suicide [27]. It seems that women usually use lower doses of drugs with latent effects which could mean that by committing suicide women try to draw attention and emotion toward themselves; however, men use higher doses of drugs which reflect their serious decisions to terminate their lives.

### Age

The highest incidence rate of completed and attempted suicides was observed in the age group of 15–24 years. However, the lowest incidence rate of attempted suicide was seen in the elderly group and the lowest rate of completed suicide was seen in the age group of 10–14 years. It seems that in Ilam province, young people, compared to the middle aged and elderly individuals are more prone to suicide using poisoning method and prefer this tool to commit suicide. One of the major contributing factors of suicide occurrence in the low age groups is probably their lower experiences during critical conditions. In fact, along with age increasing, people acquire more experiences and can exhibit more rational behaviors during critical conditions. Najafi et al., reported a high rate of suicide incidence in the age group of 20–29 years [9] and in a study conducted in the rural area of Tamil Nadu, India, the highest frequency of completed suicide due to intentional poisoning was reported in the age group of 15 to 44 years [28].In the developing countries, the highest rates of suicides usually happen among the adolescents and young married women [29]. The findings of these mentioned studies are relatively in accordance with that in the present study. In another study performed among individuals aged 15–24 years, poisoning using drugs and other substances was accounted for 5.5 and 3.6 % (incidence rate of 0.6 and 0.4 /100,000) among men, and 20.2 and 2.8 % among women (incidence rate of 0.6 and 0.1/ 100,000), respectively [30]. The incidence rate of suicide by poisoning in these studies was clearly lower than that identified by our study among those in the age group of 15–24 years, which can be related to the cultural, economical, and geographical differences between these societies. Also, in another study, the age group of 15–40 years was reported to be a high risk period for attempted suicide [31].

### Marital status

The highest incidence rate of completed and attempted suicide was identified among singles. It seems that those without a spouse experience more unstable conditions compared to married people, and may the feeling of loneliness be an associated risk factor for suicide events. Azin et al. compared the completed and attempted suicides and concluded that the highest rate of attempted suicide happened among married people, and regarding the frequency of attempted suicide events, both groups (married and single individuals) showed equal rates [32]. In another study, it was indicated that the highest rate of suicide attempts happened among women (68 %) and married individuals (57 %), respectively [33]. These findings were in contrast with our results. Pires et al., however, reported that being single was a risk factor for attempted suicide, which was in accordance with our findings [31]. These discrepancies may be related to the social, religious, economical and cultural differences among societies.

### Educational status

The highest incidence rate of attempted and completed suicides was found in the individuals studying in the secondary education level. The lowest incidence rate of completed suicide, however, was found amongst the university educated individuals. Najafi et al, reported that the highest incidence rate of suicide happened in those with diploma degree [9]. In another investigation, the highest rate of suicide events was reported among those with education level of under diploma [32]. Also, Pires et al., reported the lower level of education as one of the risk factors associated with suicide attempts [31]. In fact, it can be stated that the higher the level of education of an individual, the higher his/her level of knowledge which helps the individual to better analyze the problems and overcome hardships more rationally. Educational level, therefore, can be considered as a deterrent factor against suicide.

### Occupational status

The highest incidence rate of completed and attempted suicides was identified in unemployed and self-employed people, respectively. The lowest rate of completed and attempted suicides, however, was found among office workers and farmers, respectively. In self-employed individuals and those who cannot find a proper job appropriate to their education levels, the stress of unsteady income, the risk of unpleasant events in their jobs, loss of their jobs without having any source of monthly income, and economic failures, could be amongst the possible reasons of suicide events. Akbari Zardkhaneh et al. reported that the highest rate of suicide events was seen among unemployed and self-employed men, housewives women and high school students, respectively [34]. The highest rate of attempted suicides was also reported by another study among unemployed individuals and women [32]. These results were in accordance with our findings.

### Place of life

Attempted and completed suicides in rural areas were more frequent than urban areas. Lack of cultural, social, economical, and educational facilities and opportunities in villages compared to cities, and also transition from traditional conditions into the modernization, might be the influential factors regarding with the higher rate of suicide events in rural areas. The regression analysis showed that the probability of death due to suicide in rural areas was 1.65 times more than that in urban areas; however, inconsistent with our findings are the studies which show higher incidents of suicide using the intentional poisoning method in cities [35]. This difference may be associated with different factors affecting on selecting a suicide method by different societies either in rural or urban areas.

### Multivariate logistic regression

Multivariate logistic regression model showed that the higher the level of education, the lower the probability of suicide attempt. In another study, it was indicated that the probability of suicide attempt was higher in women with high school and academic education compared to that among men with academic education [36], which was in contrast with the findings of the present study.

Also, the probability of completed suicide in farmers was higher than that among housewives. In a study conducted in South Korea, the probability of death due to suicide using intentional poisoning was higher in divorced individuals, those with high school education and simple workers [37]. It can be concluded that those without a steady and sufficient income are more prone to different stresses compared to homemakers who are rarely engaged in financial issues. On the other hand, economical issues and addiction comprised the most important, but not significant, factors associated with the high rate of completed suicide in this study. In a review study, unemployment, addiction, and emotional disorders were considered among the risk factors of attempted suicide using poisoning [31]. Some motives such as family conflicts, humiliation, and personality disorders, were considered amongst the variables that can increase the probability of attempted suicide [34]. Economical problems and addiction can lead to family conflicts, emotional disorders, and unemployment which are known as the associated risk factors for suicide.

The probability of death due to suicide in widowed and divorced people was six times more than that among single individuals. It can be said that widowed and divorced people experienced a drastic emotional shock due to loss of their spouses, and that economic hardships along with social seclusion exacerbates their problems leading them to committing suicide. In another study, the probability rate of attempted suicide was determined as 6.55 times higher in widowed women compared to that in widowed men [36].

### Other variables

Attempted and completed suicides were most frequent in the summer and autumn than other seasons; however, their differences were not statistically significant. According to a study conducted in Iran, the highest frequency of suicide attempts among adolescents aged 12–16 was reported in winter [38] and in another investigation the highest frequency of suicides in both genders was reported in spring and autumn, respectively; however, the lowest incidence rate was reported in winter [19]. It seems that factors such as geographical conditions, seasons, and different individuals’ characteristics are influential factors associated with suicide occurrence.

The probability of death due to suicide among the individuals residing in the houses provided by the government was higher. This might be due to the fact that those who live in these types of houses don’t have free livings; however, due to the lack of data about the amount of individuals’ income in this research, it could not be concluded that those who had lived in these houses had a bad financial status. On the other hand, no statistically significant relationship was found between housing conditions and suicide. However, this factor may have an indirect effect on the family problems, unemployment, and etc.

Also, the chance of death due to suicide occurrence was higher in the extended families compared to the nuclear families. Due to the limited number of individuals in the nuclear families, their members are more intimate and in case of any family problems it can be resolved quicker within the family and this may be one of the reasons for reduction of suicide in these kinds of families. Also the highest frequency of completed suicide was seen in the Kurdish ethnicity which in fact, was due to a higher proportion of this ethnicity in Ilam province.

Although there was no statistically significant relationship between the day time of committing suicide and suicide events, the highest frequency of completed suicide was reported in the mornings which can be related to the physiological conditions of the body, such as fluctuations in the secretion of catecholamine’s and neural intermediates.

### Trend of suicide

Suicide trend during the study period (18 years) showed that females had a greater tendency to commit suicide by poisoning. The same result was reported from Kermanshah as one of the neighboring provinces of Ilam [9]; however, a Canadian study reported that males were more likely to use poisoning than females as a method of suicide [39]. This discrepancy may be related to the cultural, social, psychosocial and regional differences and also to the accessibility rate to poisons, based on genders, in these societies.

## Conclusions

The results of this large and comprehensive study, showed a high incidence rate of suicide by poisoning, especially among young and unemployed people, compared to those reported from other areas of the country, which is a concerning problem. Also females had a greater tendency to commit suicide by poisoning, and the lower level of education, the age group of 15–24 years and single individuals were more associated with suicide using poisoning. The incidence of attempted suicide in females and completed suicide in males was higher. A comprehensive plan is needed to include some educational and controlling designs for dealing of drugs and toxins to prevent the suicide events related to these methods. It also seems that the age of transition from adolescence is associated with higher risk of suicide, especially if it is accompanied with unemployment or addiction and the society needs a widespread intervention and screening for mental health in this age group.

This study had some limitations including lack of access to some variables of suicide data during 2000–2002 and 2010. There was not any data about suicide in years 2000 and 2002 and the data from 2010 were incomplete in the registration system of Ilam University of Medical Sciences. We decided not to include this information in the study. On other hand, suicide registration system monitors the registered data and removes incomplete information automatically. This monitoring is carried out by Ilam University of Medical Sciences, which leads to a reliable data at the final step. Consequently, all data except for the mentioned years were included in this study.
